# Supplementary material for: Knowledge, attitude and practices towards COVID-19 preventive measures among adults in Bhutan: A cross-sectional study
Source: PLoS One. 2022 Dec 12;17(12):e0278535. doi: 10.1371/journal.pone.0278535 (PMC9744288; doi:10.1371/journal.pone.0278535)
Supplement: S1 File — (PDF) [file pone.0278535.s001.pdf]

Section 1: Socio-demographic characteristics - Please put a tick (√) in the column where you find it relevant. And all fields are mandatory to be ticked.

1. Age in years
   - 18- 29
   - 30-39
   - 40-49
   - 50-59
   - ≥60
2. Gender
   - Male
   - Female
3. Marital status
   - Unmarried
   - Married
   - Divorced
   - Widow(er)
4. Level of education
   - Cannot read and write
   - Non-formal education
   - Monastic education
   - Primary
   - Secondary
   - Diploma
   - University education or more

5. Occupation

- Not-employed
- Student
- Private sector
- Government service
- Other*(Specify)……………………………….

6. Settlement type

- Rural
- Urban

7. Level of monthly income (NU)

- < 10,000
- 10,001-20,000
- 20,001-30,000
- 30,001-40,000
- ≥40,001

9. Sources of information on COVID-19 preventive measures [can have multiple (√)]:

- Television
- Radio
- Newspaper
- Social media [Facebook, WeChat, etc.]
- Internet
- Health care professional
- Friends and family
- Poster

Section 2: Knowledge Questionnaire- Please put a tick (√) in the column where you find it relevant. And all fields are mandatory to be ticked.

Item No.	Item	True	False	Don't Know
**K1**	Wearing a facemask can effectively prevent transmission of virus.			
**K2**	Hand hygiene (washing or sanitizing) can prevent getting COVID-19 infection.			
**K3**	Sneezing or coughing into your arm/elbow can help prevent the spread of the virus.			
**K4**	You should maintain a safe distance of at least one meter between yourself and others.			
**K5**	Virus can be transferred by shaking hands and touching your face (eyes, nose, and mouth.)			
**K6**	You should avoid going to crowded places (e.g., restaurants, religious gatherings, bars, etc.)			
**K7**	You should minimize or avoid taking public transportation.			
**K8**	The virus can stay on objects for a few days to weeks.			
**K9**	Stay home and self-isolate (avoid going to work, school and social gatherings) even if you have minor symptoms such as cough, headache and mild fever.			
**K10**	Those confirmed/suspected infections and primary contacts of the COVID-19 patients should be immediately isolated and quarantined.			
**K11**	Children and young adults need not take COVID-19 preventive measures.			
**K12**	Vaccination is important to prevent COVID-19 infection.			

Section 3: Attitude Questionnaire- Please put a tick (√) in the column where you find it relevant. And all fields are mandatory to be ticked.

Item No.	Items	Strongly Agree	Agree	Not Sure	Disagree	Strongly Disagree
**A1**	I pay close attention to the spread of COVID-19 in the country.					
**A2**	COVID-19 is an important health problem in our country.					
**A3**	My life has been disturbed by the COVID-19.					
**A4**	Following the COVID-19 preventive protocols is important in controlling the pandemic.					
**A5**	I fear of contracting COVID-19.					
**A6**	Mandatory quarantine for travelers coming from high-risk areas is an effective preventive measure.					

<u>Section 4: Practice Questionnaire-</u> Please put a tick (√) in the column where you find it relevant. And all fields are mandatory to be ticked.

Item No.	Practices of preventive behavior	Always	Often	Sometimes	Never
**P1**	I wear a facemask when I go out.				
**P2**	I wash my hands or use hand sanitizer.				
**P3**	I cover my mouth and nose with my bent elbow or a tissue when I cough or sneeze.				
**P4**	I maintain a distance of at least one meter when meeting others.				
**P5**	I avoid shaking hands or touching my face (eyes, nose, and mouth).				
**P6**	I avoid crowded places (e.g., restaurants, religious gatherings, bars, etc.) as much as possible.				
**P7**	I avoid using public transportation.				
**P8**	I clean and disinfect surfaces that are frequently, touched (door handles, faucets and phone screens).				
**P9**	I avoid going out when I have cough or fever.				
**P10**	I visit the nearest flu clinic when I have fever or cough.				
