# Supplementary material for: Knowledge, attitude and practices towards COVID-19 preventive measures among adults in Bhutan: A cross-sectional study
Source: PLoS One. 2022 Dec 12;17(12):e0278535. doi: 10.1371/journal.pone.0278535 (PMC9744288; doi:10.1371/journal.pone.0278535)
Supplement: Attachment — Submitted filename: Response to Reviewers .docx [file pone.0278535.s003.docx]

Dear Reviewers,

We would like to thank you for a thorough reading and constructive criticism of our manuscript titled “Knowledge, attitude and practice (KAP) towards COVID-19 preventive measures among Bhutanese population: A cross-sectional study”. Please find our response to reviewers’ comments in the table below. All the necessary changes and corrections in each section of the manuscript have been indicated in the “Revised Manuscript with Track Changes” file.

On behalf of my co-authors, I thank you for your consideration of this resubmission. We appreciate your time and look forward to your response.

Thanking you

Sincerely

Tshering Yangzom

Jampelyang17@gmail.com

| **SN** | **Reviewer** | **Comment** | **Response** |
| --- | --- | --- | --- |
| 1 | Journal requirement | Please provide additional details regarding participant consent. In the ethics statement in the Methods and online submission information, please ensure that you have specified what type you obtained (for instance, written or verbal, and if verbal, how it was documented and witnessed). If your study included minors, state whether you obtained consent from parents or guardians. Also, please clarify how you obtained consent from participants that were unable to read or write. If the need for consent was waived by the ethics committee, please include this information. | Line 140, Online informed consent was obtained by the enumerators before proceeding with the survey. Consent was made available in English and Dzongkha. |
| 2 |  | We note that the grant information you provided in the ‘Funding Information’ and ‘Financial Disclosure’ sections do not match.  When you resubmit, please ensure that you provide the correct grant numbers for the awards you received for your study in the ‘Funding Information’ section. | Since it was just a financial aid to help with data collection, there is no specific grant number awarded. And for the difference in the “Funding information” and “Financial disclosure” it was because there was no matching with the drop down available. |
| 3 |  | We note you have included a table to which you do not refer in the text of your manuscript. Please ensure that you refer to Table 6 in your text; if accepted, production will need this reference to link the reader to the Table. | We have changed the content of the table and reflected in the manuscript. |
| 4 | 1 | Line 100, sample size. It is unclear how the authors came up with the sample size of 1720. My calculation of the sample size with 5% Margin of error and 95% CI and an estimated response rate showed 754. I recommend that authors include the correct sample size and add a statement on why they have increased the sample size. Something like “to allow for disqualification of incomplete responses; we increased the targeted sample size to 1720”. | Line 144, subtopic “sample” and “sampling method” were clubbed together to give proper structure to the content. And A detailed sample calculation file attached. |
| 5 |  | Sampling methods and data collection heading  This section should be joined with sample size as it does not have any information about data collection, it just provides another version of sample size justification which is totally different from what has been stated in the previous section. | Line 163, new subtopic “Data collection” was added. |
| 6 |  | Line 108, authors included the selection of 86 per district and changed the sample size collection criteria. This is confusing! Please stick with sampling methods and justify its use. | Sampling procedure attached as supporting file S2 Appendix: Sampling method. |
| 7 |  | The choice of Pearson correlation is for two continuous variables and as per the study variable classification it is categorical. | Reanalysed with regression test. |
| 8 |  | 5% of the respondents are illiterate, how does this affect the results of the study considering someone else is completing the survey on their behalf. | Enumerators are trained to conduct the survey in English, Dzongkha and local dialect. |
| 9 |  | Table 2, remove no responses as it does not provide any additional information. Maybe list this table as a graph with bars the sources of information. | Table 2 replaced with Fig 1. Sources of information about COVID-19 preventive measures. |
| 10 |  | Table 3, an option of unsure should have been included in the survey questions.  Also, table 3 should include a chi test results | First of all the option “unsure” and “don't know” bear the same meaning. The study objective is to assess the levels of knowledge of the participants so that intervention could be developed accordingly. |
| 11 |  | Table 4, should include a chi test results | Separate table was added Table 5. Comparisons of knowledge, attitude and practice score among demographic variables. |
| 12 |  | Table 5, should include a chi test results |  |
| 13 |  | Line 213-220; Correlation among KAP section needs to be updated with further details as it is unclear and does not cover relevant information from the data presented. | Correlation was retested with regression analysis |
| 14 | **2** | In the title “attitudes” could be changed to “attitude” | Changed to attitude |
| 15 |  | In lines 19-20, background statements need to be more introductory about either COVID-19 or the role of Bhutanese in this pandemic. It is not preferred to highlight this " To the best of our knowledge, this is the first study performed to assess the knowledge, attitude and practice of Bhutanese population towards COVID-19 preventive measures" in this place. However, you may state the aim in a more comprehensive way. | Abstract is rewritten |
| 16 |  | In line 22, you may delete this "using a mobile application EpiCollect5 for data collection" from the abstract. |  |
| 17 |  | In line 23, " .....surveyed from all 20 districts," which sampling technique was used here? stratified sample? systematic stratified sample?. please clarify this. |  |
| 18 |  | In line 24, you may delete this: "A four section questionnaire was developed, and validated." Each participant was scored for each KAP section. " |  |
| 19 |  | In line 25, you repeat " 1708 respondents." This needs to be deleted as it is unclear. Try to focus on your findings and try to explain them clearly. |  |
| 20 |  | lines 35-39, need to be rewritten in a more intensive way and presented properly. You may present the mean COVID-19 knowledge score, attitude, and practice accordingly. |  |
| 21 |  | In lines 47 & 51, "The infection was first detected in December 2019," duplications need to be deleted. | Deleted |
| 22 |  | In line 54, "Covid-19" try to unify it all over the manuscript as COVID-19. | Line 79, Changed to COVID-19 |
| 23 |  | In line 54, "number of those infected has.." infected what? cases? people? Please clarify it. | Line 80, noted as “infected with COVID-19” |
| 24 |  | In line 69, "however instituted in the early months of 2020.", delete however.. what months? Please summarize and cite it. | Line 103, “by mid-January” and cited accordingly |
| 25 |  | in line 72, how about other preventive measures? Vaccine? % of vaccinated people? try to add these. | 87.2% of total population is vaccinated as of 29.07.22 |
| 26 |  | lines 77-80 could be shorted and merged. | Necessary changes were made. |
| 27 |  | In line 89, change "nationals" to "people". | Line 132, “nationals” to “people” |
| 28 |  | study setting needs to be stated clearly; e.g., which 20 districts? any cities? Why did you choose these 20 locations? " | Bhutan has only 20 districts and since this is a nationwide survey, all the districts were included. |
| 29 |  | In sample size, what is the total population of Butane? How did you assume without a total targeted population number? Please try to correct this. | The total population is taken from the Population and Housing census of Bhutan 2017: 727,145 |
| 30 |  | Is Epicollect5 the only platform available? Did all the butane have it ? Why do you just use this platform? | No, Epicollect5 is not the only platform available. Since the app is free of cost and downloadable in any android or IOS. We used this platform because of the following reasons:   1. User friendly. 2. Free of cost. 3. Can track enumerators as the app is linked with a GPS system. |
| 31 |  | In line 125, "Hence, the questionnaire was tailor-made to best suit the Bhutanese population." How did you confirm this? Did you pilot the Survey? If so, how? Which language did you use? If you pilot, then what is the alfa Cronbach score? | Line 209, The reliability of the instrument was pretested among 30 participants who shared similar characteristics as the subjects under the study. They were selected through convenience sampling. The internal consistency reliability (Cronbach’s α) value for the instrument was 0.88. Questionnaire was developed in English and later translated into the national language *Dzongkha*.  Questionnaire attached as supporting file S3 appendix: Survey questionnaire. |
| 32 |  | in line 130, " assess the participants' awareness." How to assess participants' awareness or knowledge? Please clarify this and correct it. | Participants' knowledge was assessed by a set of 12 questionnaires with answer options of “True, False and Don’t know”. |
| 33 |  | In lines 136 and 137, " fourth section is constituted of 10 items (P1-P10), with four-point Likert scale responses (always=4, never=1)". This for what? What exactly is this evaluation? | Line 205,”Fourth section constituted of 10 items (P1-P10), with four-point Likert scale responses (always=4, never=1) to assess the participants' COVID-19 preventive practices. The total score in this section would range from 10-40. The higher scores correlate with better preventive actions being taken.” |
| 34 |  | line 156, "response rate of 99.3%" How did you calculate this? If there is a withdrawal and a refusal rate? try to state that and the reasons behind it. | A total of 1708 participants from a sample size of 1720 were surveyed, hence a response rate of 99.3%.  12 households could not be surveyed in an area due to the increasing number of COVID-19 which led to the region being under lockdown during the survey period. |
| 35 |  | In lines 156–160, do these findings correlate with the Butanes census? Alternatively, there is no random or stratified selection. | As per 2017 population and housing census, 47.7% are female |
| 36 |  | In line 220, abbreviations for the tables need to be stated and footer needs to be added. | KAP: Knowledge, attitude and practice. |
| 37 |  | Table 6 needs to be represented with all the details. Where is the P-value? | Correlation was retested. |
| 38 |  | Regression could be a better explanation of these results if you did it. |  |
| 39 |  | Limitation section to be added. | Limitation section was added. |
| 40 | **3** | Methods, result and discussion  a. Under demographic characteristics: Good to give information regarding the total population demographics on married, younger than 40 years of age, occupational distribution, and rural vs. urban population so we can see the sample representation of this study. | Detailed information on various demographic characteristics is shown in table 1. |
| 41 |  | Methods, result and discussion  b. Practice of COVID-19 preventive measures- it is challenging to assume practice based on how study participants answered few questions. Usually there is inflation of actual practice when compared to an observed practice. Ideally, observation of practice should have been part of the study to confirm practice rather than simple survey. To support that, is there any study in the same community that researchers can site to corroborate the practice of hand hygiene and use of facemask? It is helpful to see those data. | It would have been better if we could actually observe preventive practice measures adopted by the general people, however, due to the time and human resource constraints we were only able to survey on the measures undertaken. We regret to state this as one of the limitation of this study. |
| 42 |  | Methods, result and discussion  c. Another objective indirect surrogate marker for good attitude and practice would have been vaccine acceptance rate. Assumption is in society with high KAP of COVID-19, actual vaccine acceptance will be higher. Adding this on the discussion and examining its statistical correlation with attitude and practice might give another direction of examination of the data. | Since the study objective was to provide a baseline on KAP level, and the vaccination program was still in its initial phase when we started the study, it was too early for us to integrate it in our study. |
| 43 | **4** | The details of clinical presentation of the first case are irrelevant in the instant manuscript and could be omitted (lines 73-75) | omitted |
| 44 |  | Did educational status determine the level of knowledge, etc. That might need address. | Relationship was reexamined with regression test. |
| 45 |  | Did the various collected variables correlate with the KAP about COVID vaccines. Normally these sociodemographic features factors do affect.  Also the effect of age should be teased out. Younger population has greater access to modern modes of communication and it may be worthwhile to look at the association, if any. |  |
| 46 |  | Some discrepancy between the numbers in table 5 and those depicted here (line 208-210). Needs to be ironed. | Always and often scores were combined for percentage of good practice. |
| 47 |  | Grammatical error (P8 table 5) | Correction is made. |
| 48 |  | Some discrepancy in numbers needs to be clarified. For example 48% always avoid going out if they feel unwell with fever or cough. However in P10, 55.6% ALWAYS visit a flu clinic when confronted with such symptoms. The numbers do not match and might need a relook by the authors. | 48% avoid going out unnecessarily; however, the public are notified to visit the nearest flu clinic in case of prolonged duration of symptoms or worsening signs and symptoms. |
| 49 |  | The scoring system is not clear and we need to know the score classification that was attributed as low, high or average. For my understanding 89% is fairly high. | It's true that 89% is fairly high, however what we intend to reflect is the average accuracy rate (average of true response for all 12 questionnaires of the knowledge section) |
