# Supplementary material for: Knowledge, attitude and practices towards COVID-19 preventive measures among adults in Bhutan: A cross-sectional study
Source: PLoS One. 2022 Dec 12;17(12):e0278535. doi: 10.1371/journal.pone.0278535 (PMC9744288; doi:10.1371/journal.pone.0278535)
Supplement: Attachment — Submitted filename: Response to Reviewers .docx [file pone.0278535.s004.docx]

Dear Reviewers,

We would like to thank you for a thorough reading and constructive criticism of our manuscript titled “Knowledge, attitude and practice towards COVID-19 preventive measures among adults in Bhutan: a cross-sectional study”. Please find our response to reviewers’ comments in the table below. All the necessary changes and corrections in each section of the manuscript have been indicated in the “Revised Manuscript with Track Changes” file.

On behalf of my co-authors, I thank you for your consideration of this resubmission. We appreciate your time and look forward to your response.

Thanking you

Sincerely

Tshering Yangzom

jampelyang17@gmail.com

| **SN** | **Reviewer** | **Comment** | **Response** |
| --- | --- | --- | --- |
| 1 | Journal requirement | Please review your reference list to ensure that it is complete and correct. If you have cited papers that have been retracted, please include the rationale for doing so in the manuscript text, or remove these references and replace them with relevant current references. Any changes to the reference list should be mentioned in the rebuttal letter that accompanies your revised manuscript. If you need to cite a retracted article, indicate the article’s retracted status in the References list and also include a citation and full reference for the retraction notice. | In the reference we removed reference 12 where we cited the Ministry of Health’s national situational update on COVID-19 because the latest update on the website was of September 19, whereas the latest updated count on WHO dashboard was dated November 11, 2022.  Reference number 25 was also removed as the same article was cited twice.  Reference number 4, 16, 20 and 28 was removed as the citation was edited while revising the manuscript.  Some relevant studies are cited in the revised manuscript hence, reference number 13, 14, 15, 16, 27 and 28 are added. |
| 2 | Additional Editor Comments | Please revise this statement in page 3, “According to the WHO, as of 11th April 2022, there were 497,057,239 confirmed cases of COVID-19 globally with a death count of 6,179.104 (5)”: please update the counts as of the date of submission, and revise ref 5, the death count was wrong. | Confirmed cases of COVID-19 and deaths counts for both globally and Bhutan are updated as per the latest (11 November 2022) record on the WHO Coronavirus (COVID-19) Dashboard. |
| 3 |  | Page 6, the last paragraphs: “..and the second, third and fourth section consisted of 28 questions: 12 knowledge-based, 6 attitude-based and 10 practice-based respectively’ are confusing; please state separately how many questions are there in the second, third and the fourth sections. We now understand the second section has 12 questions, so no need to repeat that in the subsequent paragraph. Avoid using ‘question’ and ‘item’ interchangeably, use one term consistently. | The second paragraph of ‘Study Instrument’ was rephrased to make the sentence more meaningful and term ‘item’ was used for uniformity. |
| 4 |  | The scoring for the second section needs rethinking as it does not make logical sense. You gave 1 score for “don’t know”, 2 for “false” and 3 for “true”. False is the opposite of true, so if someone provides a false answer they deserve the lowest score, while ‘don’t know’ is an honest of deliberation of not knowing. In the society people who say they don’t know are safer than those who spread false information. | To make the scoring for the knowledge section more logical, the scoring system was changed to 1 point for correct response and 0 points for incorrect or “don’t know” response. |
| 5 |  | Some acronyms like ‘S-CVI’, ‘I-CVI’ and ‘CVR’ have not been spelt out on first use. Also in acknowledgement section hospital or department name like JDWNR hospital or VPDP need to be spelt out. | All the acronyms are spelt out where ever necessary. |
